# An evidence-based framework to measure quality of allied health care

**DOI:** 10.1186/1478-4505-12-10

**Published:** 2014-02-26

**Authors:** Karen Grimmer, Lucylynn Lizarondo, Saravana Kumar, Erica Bell, Michael Buist, Philip Weinstein

**Affiliations:** 1 International Centre for Allied Health Evidence, The University of South Australia, City East Campus, Adelaide 5000, Australia; 2 University of Tasmania, Sandy Bay Campus, Hobart, Tasmania 7001, Australia; 3 Barbara Hardy Institute, University of South Australia, City West Campus, Adelaide 5000, Australia

**Keywords:** Allied health, Therapy, Quality constructs

## Abstract

**Background:**

There is no standard way of describing the complexities of allied health (AH) care, or its quality. AH is an umbrella term which excludes medicine and nursing, and variably includes disciplines which provide therapy, diagnostic, or scientific services. This paper outlines a framework for a standard approach to evaluate the quality of AH therapy services.

**Methods:**

A realist synthesis framework describing what AH does, how it does it, and what is achieved, was developed. This was populated by the findings of a systematic review of literature published since 1980 reporting concepts of quality relevant to AH. Articles were included on quality measurement concepts, theories, debates, and/or hypothetical frameworks.

**Results:**

Of 139 included articles, 21 reported on descriptions of quality potentially relevant to AH. From these, 24 measures of quality were identified, with 15 potentially relating to what AH does, 17 to how AH delivers care, 8 relating to short term functional outcomes, and 9 relating to longer term functional and health system outcomes.

**Conclusions:**

A novel evidence-based quality framework was proposed to address the complexity of AH therapies. This should assist in better evaluation of AH processes and outcomes, costs, and evidence-based engagement of AH providers in healthcare teams.

## Background

Accurate and appropriate measures of the quality of care provided by any health professional are essential to provide evidence that care is the best it could be [1,2]. For over two decades, the healthcare industry internationally has sought ways of describing the nature, volume, complexity, costs, and outcomes of its services, to deal with increasing consumer demand, ballooning healthcare costs, and tightening financial constraints [3,4]. Standardized assessments of healthcare performance have been widely implemented internationally via accreditation standards, benchmarks, and/or key performance indicators [5-8]. However, the challenging issue remains of what does ‘healthcare quality’ look like? This dilemma is described by Davila [9], who observed that “*For many, quality healthcare is like beauty or pornography—they know it when they see it but they just can’t define it. Yet, a widely accepted and specific definition of quality healthcare is required for its assessment and promotion, and a lack of this definition makes these impossible. The sum and substance, then, is ‘What is an acceptable and specific definition of quality healthcare?’*” (p. 84).

There is already a considerable body of evidence describing a range of measures purporting to relate to healthcare quality, however, most of them relate to hospitals [10-12], medicine, or nursing [13-15]. None are specific to allied health (AH) and thus, to date, the complexities of what AH does, how they deliver care, and what is achieved, remain largely uncaptured. AH is an umbrella term used to describe a range of health disciplines and ancillary services (excluding medicine and nursing) which provide therapy, organizational, and/or scientific services. The complexity of AH is such that there is no standardly-agreed definition. It is usually described by discipline lists and/or tasks, which can vary between countries, government bodies, industry, healthcare settings, and training institutions [16-21].

The international literature lists common AH disciplines as audiology, dietetics and nutrition, occupational therapy, orthoptics, orthotics and prosthetics, physiotherapy (physical therapy), podiatry, psychology, radiography, speech pathology, and social work. The range of tasks undertaken by these AH disciplines [20,22] are summarized in Table 1. In general, dietetics and nutrition, occupational therapy, physiotherapy, podiatry, psychology, and speech pathology are primarily considered therapy (treatment) services; audiology, orthoptics, and radiology are primarily considered diagnostic services; orthotics/prosthetics is primarily an assessment and manufacturing service; and social work provides primarily organization and counseling services. However, task sharing and overlap is often found between AH disciplines, depending on patient need, service location, availability, access and purpose, and clinician expertise [20,22]. Moreover, AH disciplines commonly work in multidisciplinary teams [23] in which task sharing is common. AH services are available in the public and private sectors, and across settings (acute hospitals, sub-acute, community, rehabilitation, and primary healthcare). AH services optimize functional capacity and quality of life throughout the lifespan [24,25].

**Table 1:** The type of activities undertaken by common allied health (AH) disciplines

	**Therapy (treatment)**	**Assessment**	**Diagnosis**	**Counseling**	**Education**	**Manufacture/prescription**	**Organization**
Audiology		Peripheral	Core		Peripheral	Peripheral	
**Dietetics and nutrition**	Core	Peripheral		Peripheral	Peripheral		Peripheral
**Occupational therapy**	Core	Peripheral		Peripheral	Peripheral	Peripheral	Peripheral
Orthoptics	Peripheral	Peripheral	Core	Peripheral	Peripheral	Peripheral	
Orthotics and prosthetics	Peripheral	Core			Peripheral	Core	Peripheral
**Physiotherapy**	Core	Peripheral	Peripheral	Peripheral	Peripheral	Peripheral	Peripheral
**Podiatry**	Core	Peripheral	Peripheral	Peripheral	Peripheral	Peripheral	
**Psychology**	Core	Peripheral	Peripheral	Peripheral			
Radiography		Core					
**Speech pathology**	Core	Peripheral	Peripheral	Peripheral	Peripheral		
Social work		Peripheral		Core	Peripheral		Core

Bold: Disciplines primarily undertaking therapy (treatment) which are the focus of this review; Core: Core activity/ies of the discipline; Peripheral: Peripheral tasks.

An important key point of difference in measuring AH quality (compared to medicine and nursing) is service delivery. AH services (particularly the therapies) are generally provided to patients in episodes of care, not in the medical model of ‘occasions of service’ [26]. To manage the presenting problem, multiple AH tasks may be undertaken within one patient-contact (occasion of service), as well as over an episode of care (multiple linked occasions of service). However, there is variable quality research evidence for the choice of assessments, diagnostic procedures, and interventions used in AH occasions of service or episodes of care [20,24,27-29].

The percentage of the 2011 global health workforce attributed to AH was 5% to 11%, which is similar to medicine but smaller than nursing [30]. In the most recent Australian health workforce statistics [31], there were 57,019 medical practitioners, 65,284 AH professionals (not including pharmacists or complementary medicine), and 202,735 registered nurses. Therefore, AH services represent a significant part of the Australian workforce, and as such, robust measurement of AH quality is urgent.

The aim of this systematic review was to distil the literature to conceptualize and inform measurement of quality of AH services. The findings from the review led to the development of a novel evidence-based framework to measure AH quality.

## Methods

### Research design

A systematic literature review framed in a realist synthesis model.

### Search strategy

Structured library database searches were conducted to identify peer-reviewed articles related to healthcare quality constructs and quality measurement relevant to AH.

### Inclusion/exclusion criteria

Studies which reported on health quality measurement concepts, theories, and hypothetical frameworks were included. Full text English-language peer-reviewed journal articles were considered, including reviews, experimental studies, observational studies, case studies, commentaries, concept papers, and validation studies. Conference abstracts, abstracts only of published literature, articles in languages other than English (without available translation), and grey (non-peer-reviewed) literature, were excluded.

### Library databases

OVID, Medline, CINAHL, Ageline, AMED, EMBASE.

### Keywords

AH descriptors (allied health or physiotherap* or “physical therap* or occupational therap* or speech therap* or “speech patholog* or diet* or nutrition* or social work* or podiatr* or or orthotist or prosthetist or psycholog*); quality (quality of healthcare or healthcare quality or health service quality); measure* (measurements or outcomes or outcome measurement or process assessment or process measurement or health outcome measures). Synonymous terms and related Medical Subject Headings (MeSH) was used to expand the search as appropriate within individual databases.

### Pearling

Reference lists of included articles were searched for relevant references not found in the library database search.

### Date range

Reflecting the emergence of the healthcare quality evaluation movement, publications since 1980 were sought, although the search strategies did not set a date limit. This allowed seminal papers written prior to 1980 to be identified and included, as relevant. Seminal papers were those which were regularly cited in subsequent research, and which had a significant impact on the evolution of quality care research.

### Quality assessment

The study design of included literature was determined using the National Health and Medical Research Council (Australia) intervention hierarchy [32]. Methodological quality was not assessed as per the review aims.

### Data extraction

Data was extracted on author, year, country, study hierarchy, quality descriptors, condition/patient group (if relevant), and AH discipline. Articles of interest in this review primarily dealt with quality measurement concepts, theories, debates and/or hypothetical frameworks. The included evidence was classified into four streams: i) conceptual and/or theoretical frameworks for evaluating health service quality; ii) service quality data items; iii) patient assessment of service quality; and iv) reporting mechanisms for service quality.

### Data synthesis

**Figure 1:**
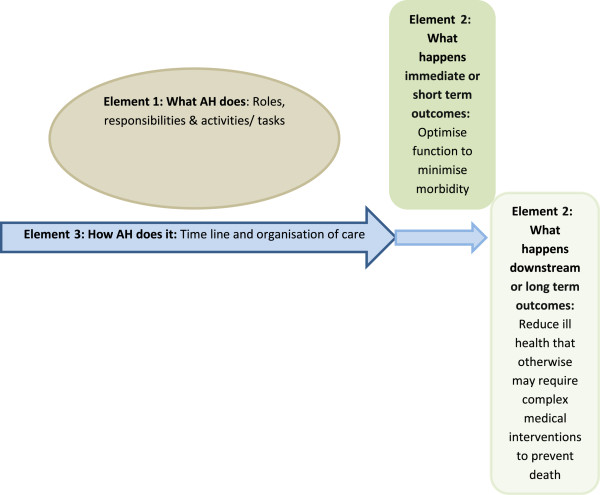
Step 1 in the realist synthesis theoretical framework.

The review was framed in a realist synthesis model, which used a three-element theoretical framework describing AH service delivery [33] (Figure 1).

#### *Element 1*

‘What AH therapy does’ considered AH roles, responsibilities and tasks [20,22] (Table 1). The activity list is not exclusive, nor may it be appropriate in individual circumstances; however, this table demonstrates the complexity and overlap of activities, specifically relevant to AH therapy disciplines (bold).

#### *Element 2*

‘What happens’ considered outcomes from the AH disciplines (Table 1). Despite task differences, a common AH outcome is optimization of function [24,25]. This may be a short-term effect of minimizing/preventing morbidity and handicap, and may well have a downstream effect of reducing further ill-health, major health events, or even death (such as podiatric diabetic ulcer management, which significantly reduces the risk of gangrene and subsequent limb amputation) [34].

#### *Element 3*

‘How AH does it?’ describes organization of care in terms of occasions of service and episodes of care [26].

## Results

### Literature base

**Figure 2:**
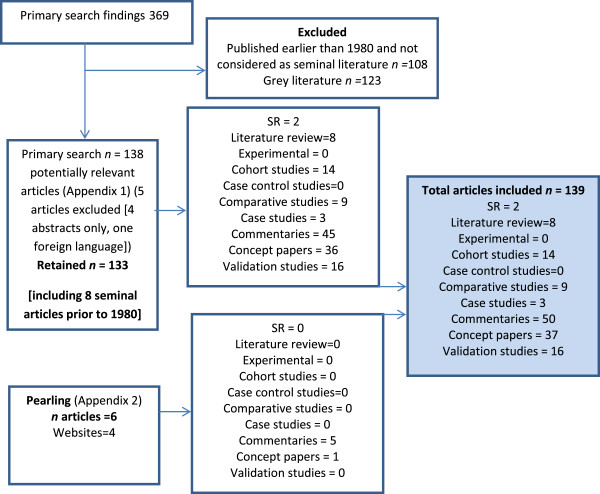
Consort diagram considering all included articles.

Potentially relevant articles (n = 369) were identified from searching electronic databases, and 6 additional potentially relevant articles were identified via pearling. Of the potentially-relevant articles, 108 were non-seminal articles published before 1980 and 123 were grey literature and were hence excluded, leaving 138 potentially relevant papers from electronic databases (Additional file 1). Five more articles were subsequently excluded as they were either available in abstract form only or written in a language other than English; 133 articles from electronic databases were retained. An additional 6 articles were found from pearling, and all were considered relevant to the review. Therefore, a total of 139 articles was included. Within this list there were 8 seminal references which had been published prior to 1980 and identified in the database search [35-41]. They were included in this review as their impact on healthcare quality research was validated during pearling. Figure 2 outlines the consort diagram.

### Hierarchy of evidence

The included studies comprised commentaries and opinion (36.4%), theoretical or debate papers (26.4 %), validation studies (11.4%), cohort studies (10%), comparative studies (6%), non-systematic literature reviews (6%), case studies (2%), and systematic literature reviews (1%).

### Discipline focus

Only two articles (1.4%) dealt directly with AH; a patient satisfaction survey with orthotics and prosthetics [42] and a letter to the Editor regarding the value of AH students in an outpatient teaching clinic [43]. The majority (79.3%) of papers were written from the perspective of doctors and nurses in acute health services; the remaining papers reflected perspectives of managers, policy makers, funders, Information Technology personnel, statisticians, and service quality managers.

### Literature classification

The included literature reflected four main streams, as identified in the paper’s title, abstract and/or purpose. These streams were classified as: i) measurement of quality within a service (66 papers); ii) concepts and frameworks for evaluating the quality of a service (37 papers); iii) measurement of quality of a service by patients (19 papers); and iv) reporting service quality to stakeholders (17 papers). The classifications for the included papers are provided in Additional file 1.

### The focus of this paper

**Figure 3:**
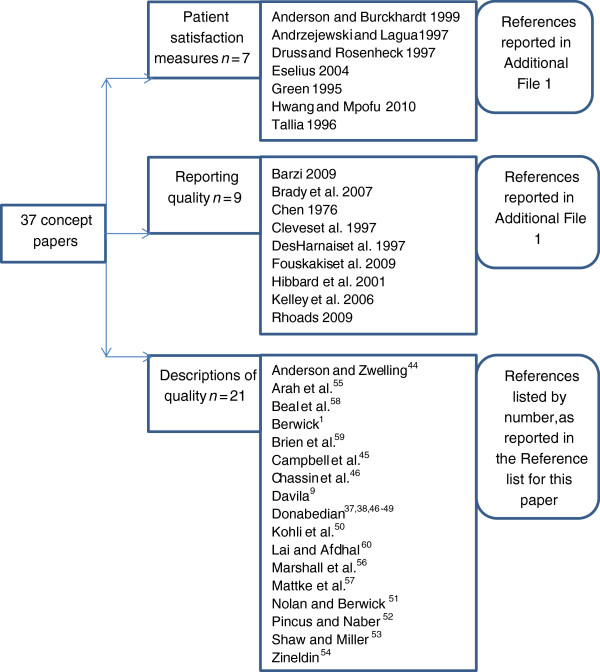
Conceptual measures of quality potentially relevant to allied health.

This paper focused on the stream of literature that we believed could provide guidance regarding allied health quality measures, these being the concept papers. These papers reflected three main areas: concepts in patient satisfaction measures (n = 7), concepts in reporting quality (n = 9), and concepts in describing quality (n = 21), as listed in Figure 3. The ‘descriptions of quality’ concept papers formed the basis for the discussions presented in this paper. Specifically, this literature subset comprised: position statements or academic debate (n = 13) [37,38,44-54]; comparative reports of international quality definitions (n = 3) [55-57]; reflections on what health quality could mean (n = 2) [58,59]; letters to the Editor (n = 2) [9,60]; and one presidential address [1].

### Seminal quality frameworks

Commonly underpinning this subset of the concept literature was the Donabedian quality framework of structure, processes and outcomes [37,38,47,48], and/or pillars of quality (efficacy, effectiveness, efficiency, optimality, acceptability, legitimacy, and equity) [49]. The notion of maintaining high levels of both technical and functional quality grew from these pillars [44]. Technical quality relating to Donabedian pillars of efficacy, effectiveness, efficiency, and optimality refers to the product and its cost (e.g., length of stay, infection, mortality rates), whereas functional quality refers to service delivery issues (Donabedian pillars of acceptability, legitimacy, and equity), and includes customer satisfaction. Adaptations of these pillars have been proposed: Kohli et al. [50] described core quality measures of cost information, clinical outcomes and patient satisfaction. Campbell et al. [45] suggested dimensions of quality of care of access and effectiveness to answer the questions of: ‘*Do users get the care they need, and is the care effective when they get it?’* (p. 1611). Accessibility was related to geographic access, affordability and availability, and outcomes of effectiveness and accessibility relate to health status, user evaluation, clinical and interpersonal care. Shaw and Miller [53] proposed multifactorial domains for disease outcomes (clinical, humanistic, and economic). The clinical domain was divided into healthcare systems, healthcare needs, and individuals. Zineldin [54] proposed multiple measures of quality: object (technical quality), process (functional quality), infrastructure (basic resources needed to perform healthcare services), interaction (quality of information exchange), and atmosphere within which care is provided. Beal et al. [58] prioritized quality domains in pediatrics as i) effectiveness, ii) timeliness, iii) patient-centeredness, and iv) safety. This paper also presented a framework considering pediatric quality of care as staying healthy, getting better, living with illness, and end of life.

This subset of literature also reflected debate on the relationship between measures of quality and evidence-based practice: Berwick [1] challenged The Society of Medical Decision-Making with the notion of integrating patient-centered care, evidence-based practice, and cost containment, in order to deliver the right care to the right person at the right price at the right time. Chassin et al. [46] presented the concept of underuse, overuse, and misuse of care, compared with agreed quality benchmarks of care. Davila [9] suggested that quality should be an integration of measures of treatment effectiveness, evidence-based practice, and patient satisfaction. The ‘all or none’ model [51] presents a measureable approach to putting evidence into practice for every patient, every time; there is, however, debate about its achievability [61]. Brien et al. [59] discussed integrating evidence-based practice and performance indicators for specific health conditions. Their premise was that evidence should be distilled into clinical practice guidelines, and interpreted as performance indicators. These could then generate data on system performance which supports the development of quality improvement programs. Pincus and Naber [52] presented elements of a quality strategy for mental healthcare which included a common set of quality measures, methods to collect and report on core data, and validated assessment instruments (p. 609). Finally, Lai and Afdhal [60] reported on the concept of ‘if, then’ indicators (“*if’ characterizes the eligible patient population, and ‘then’ describes the care that should be given?*”) (p. 650).

This subset of the concept literature also identified that, over the past decade, the Organization for Economic Cooperation and Development (OECD) has considered healthcare quality from the broader societal and public health approach towards health determination, and the individual clinical/technical view regarding individual patient needs. The OECD has sponsored inter-country round-table discussions regarding quality domains. Arah et al. [55] reported these discussions and outlined multiple quality domains (acceptability, accessibility, appropriateness, care environment and amenities, expenditure, governance, competency or capability, continuity, patient-centeredness, effectiveness, improved care, clinical focus, efficiency, safety, sustainability, and timeliness). Marshall et al. [56] reported on the concept model of the continuum of care starting from population-based health services (health promotion), then encompassing preventative care and ending at personalized medical care (diagnosis and treatment).

### Development of a novel AH quality framework

**Figure 4:**
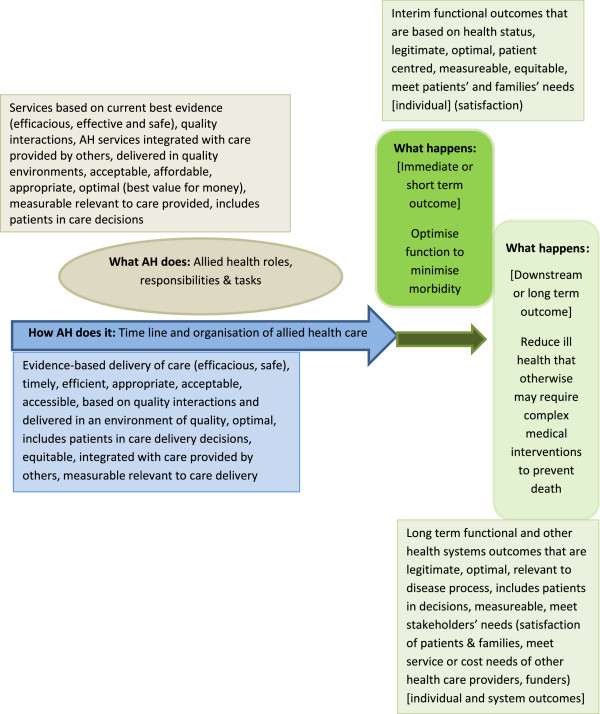
Step 2 of realist synthesis: Population of the theoretical model with literature findings.

In its entirety, the evidence-base identified in this review was not immediately generalizable to measuring quality specifically of AH therapy services. However, the realist synthesis approach allowed us to populate our framework describing the complex, episode-of-care nature of AH therapy services, with the current quality concept evidence-base, and thus to develop a framework for evaluating AH therapy quality (Figure 4). We consequently suggest evidence-based measures to assess the quality of AH therapy services (Table 2). When proposing measures of patient-centeredness, we employed all three approaches outlined in the literature (patient engagement in care decisions, patient satisfaction with care, and outcomes) [62]. Patient engagement in AH care decisions, and patient satisfaction with care and outcomes, is not just about determining what care is preferred (and why), but also about how it is provided (its frequency and duration, e.g., within an episode of care) [26] and valued endpoints of care. It could also include measures of the way information is exchanged with patients to assist them to make care decisions, the way their choices are incorporated into treatment decisions, efficacy of treatment options presented, and the quality of both the interaction and the information exchanged [1,45,54,59].

**Table 2:** Proposed quality measures relevant to AH therapy services

	**What AH does?**	**What happens?**	**How AH does it?**	**What happens next?**
Efficacious treatments [1,45,47,49,59]	×		×	
Effective treatments [9,44,45,49,55,58]	×			
Safe treatments [46,55,58]	×		×	
Best practice care occasions or episodes [59]			×	
Quality interactions with others [45,54]	×		×	
Integrated care with other care [45,54]	×		×	
Quality environments of care [55,54]	×		×	
Acceptable [44,49,55]	×		×	
Affordable [45,50]	×		×	
Value for money (optimal) [45,60]	×	×	×	×
Patient satisfaction with care delivery [62]		×	×	×
Patient satisfaction with care [62]	×			
Patient satisfaction with outcomes [62]		×		×
Timely [55,58]			×	
Efficient [44,49,55]			×	
Appropriate [55]	×		×	
Equitable [44,49]	×	×	×	×
Legitimate [44,49]	×		×	
Reflect societal health status measures [56]				×
Reflect individual health status measures [56]		×		×
Measurable relevant to care provided [44]	×	×		×
Measurable relevant to care delivery [44]		×	×	×
Measurable relevant to health outcomes [44]		×		×
Includes patient in care decisions [1,55,58]	×		×	

## Discussion

The proposed evidence-based quality measurement framework (Table 2) is the first of its kind, to the authors’ knowledge, that can be applied to AH therapies. To make a start on measuring AH therapy quality, we suggest that the most common quality elements across our framework (optimality [value for money], patient satisfaction with care delivery, and equity) could be developed into ubiquitous measurable data items and performance indicators for all AH therapy services. Once these items and indicators are in place, and barriers to their uptake identified, benchmarking could occur between the same therapy discipline in different healthcare settings. This would provide unique evidence- and performance-based information with which to then consider other important aspects of AH quality.

Our framework requires development in terms of data items and performance indicators, relevant to individual AH therapies, local contexts, patients, funders, and other healthcare providers. For instance, while efficacy might be readily addressed by an organization’s mission statement (usually a commitment to providing evidence-based care supported by access to a comprehensive medical library), how ‘timeliness’ is measured requires input from end-users of AH services because of its multiple meanings [3]. ‘Timeliness’ data could reflect the referral pathways by which patients are referred to AH therapies, patient eligibility and expectations, waiting time and the opportunity cost of this, patient and family perspectives on service equity and access, and optimality of services in the short and longer term. To arrive at this next stage will require the use of different lenses and research methodologies to take account of the complexities and activities of each AH service.

At the end of it all, quality evaluation of AH care should provide a means to identify the right care provided to the right patient, at the right time (particularly throughout an episode of care), the nature and delivery of the care, integration with care provided by others, and communication between healthcare providers and patients [2]. Thus, quality measures should reflect the perspectives of the many ‘stakeholders’ who “*will know quality when they see it*” ([9], p. 84).

Of note from this review, is that medical and nursing professions appear to not yet have the correct definition or measurement of quality, either. For instance, the effectiveness of medical care is currently determined not by the quality of ‘doctoring’ but generally by the use of processes or interventions [63]. This is underpinned by a growing body of literature on the failure of the medical profession to implement or apply proven interventions when they are indicated [64]. Thus, it may be that by taking an evidence-informed approach to quality measurement, and learning from current medicine and nursing evidence regarding quality measurement, AH disciplines might position themselves well to identify what ‘quality’ really means to its stakeholders [58,65].

## Conclusions

This review found no current measure of healthcare quality specific to AH therapy services. Differences within, and between, AH disciplines, and with medicine and nursing, mean that a novel lens should be applied to develop appropriate quality measures for AH. Unless the complexity of AH activities, responsibilities, and service-delivery patterns can be expressed in service-specific ways, the value of AH therapy services will be overlooked when healthcare quality is evaluated and reported.

Our novel quality framework (Figure 4 and Table 2) identified 24 quality measures relevant to at least one aspect of AH therapy. Three quality measures were common (optimality, patient satisfaction with care delivery, and equity). AH ‘stakeholders’ (policy-makers, researchers, clinicians, managers, and patients) can now develop these into practical AH-specific descriptions of service quality; this can then be built on to develop and refine other quality measures relevant to other AH tasks and disciplines. Our novel quality framework will contribute to specific AH performance assessment to improve the delivery and acceptability of AH services. This will contribute to better implementation of evidence-based decisions, more consistent consideration of patient choices, and improved health outcomes.

## Competing interests

The authors declare that they have no competing interests.

## Authors’ contributions

KG and LL, assisted by SK, undertook the extensive literature review, constructed the realist synthesis framework and populated the framework with early literature synthesis. Advice was sought after the initial framework was constructed from EB and MB, in terms of literature classification and interpretation, as they represent non-allied health, health researchers, whose validation of our allied health thinking was essential to the arguments we present in the paper. PW took a broader view of the manuscript after it was drafted, and provided significant input into the ‘so what’ of the findings. His input assisted us in developing Figure 4. He also took a large part of the editing. All authors read and approved the final manuscript.

## Supplementary Material

Additional file 1 Included articles and their classifications. Click here for file
