# Supplementary material for: An evidence-based framework to measure quality of allied health care
Source: Health Res Policy Syst. 2014 Feb 26;12:10. doi: 10.1186/1478-4505-12-10 (PMC4015500; doi:10.1186/1478-4505-12-10)
Supplement: Additional file 1 — Included articles and their classifications. [file 1478-4505-12-10-S1.docx]

**Additional File 1.** Included literature

| **Reference** | **Classification** |
| --- | --- |
| AHRQ Research Activities. Journal supplement explores lessons to be learned in health care quality and disparities from AHRQ's first national reports. *AHRQ Res Activ* 2005; 298: 22. | reporting |
| AHRQ Research Activities. AHRQ launches new Web-based tool for States to measure performance on the quality of health care. *AHRQ Res Activ* 2006; 307: 18. | reporting |
| AHRQ Research Activities. New Web tool provides samples of report cards on health care quality. *AHRQ Res Activ* 2007; 323: 16. | reporting |
| AHRQ Research Activities. Improvements are needed to better measure mental health care quality. *AHRQ Res Activ* 2010; 357: 5. | quality in service |
| Adinolfi P. Total quality management in public health care: a study of Italian and Irish hospitals. *Total Qual Manage Bus Excel* 2003; 14: 141-150. | concept |
| Anderson EA, Zwelling LA. Strategic service quality management for health care. *Am J Med Qual* 1996; 11: 3-10. | concept |
| Anderson K, Burckhardt C. Conceptualization and measurement of quality of life as an outcome variable for health care intervention and research. *J Adv Nurs* 1999; 29: 298-306. | concept |
| Andrzejewski N, Lagua RT. Use of a customer satisfaction survey by health care regulators: a tool for total quality management. *Public Health Rep* 1997; 112: 206-210; discussion 211. | quality in service |
| Anonymous. Health Care Marketers Struggle to Define 'Quality'. *Market New* 1990; 24: 21. | quality in service |
| Arah OA, Westert GP, Hurst J, et al. A conceptual framework for the OECD Health Care Quality Indicators Project. *Int J Qual Health Care* 2006; 18: 5-13. | concept |
| Barzi A. *Quality in healthcare organizations: Its meaning and measurement.* US: School of Public Health, The University of Texas, 2009; 70. | concept |
| Beal AC, Co JPT, Dougherty D, et al. Quality measures for children's health care. *Pediatr* 2004; 113: 199-209. | concept |
| Bellinger AC. An examination of some issues pertinent to evaluation research and the assessment of health care quality. *Am Nurse Assoc Pub* 1976; (G-124): 115-127. | quality in service |
| Benda C. Health care quality. How we measure, define, and refine it. *Minnesota Med* 1989; 72: 397-400. | reporting |
| Berwick DM. Toward an applied technology for quality measurement in health. *Med Decis Mak* 1988; 8: 253-258. | quality in service |
| Bierman AS, Lawrence WF, Haffer SC, et al. Functional health outcomes as a measure of health care quality for Medicare beneficiaries. *Health Serv Res* 2001; 36: 90-109. | concept |
| Bost JE, Thompson JW, Shih S, et al. Differences in health care quality for children and adults under managed care: Justification for separate quality assessments? *Ambul Pediatr* 2002; 2: 224-229. | quality in service |
| Bowers MR, Kiefe CI. Measuring health care quality: Comparing and contrasting the medical and the marketing approaches. *Am J Med Qual* 2002; 17: 136-144. | quality in service |
| Brady J, Ho K, Kelley E, et al. AHRQs national healthcare quality and disparities reports: An ever-expanding road map for improvement. *Health Serv Res* 2007; 42: xi-xxi. | quality in service |
| Brien SE, Dixon E, GhaliWA. Measuring and Reporting on Quality in Health Care: A Framework and Road Map for Improving Care. *J Surg Oncol* 2009; 99: 462-466. | concept |
| Campbell SM, Roland MO, Buetow SA. Defining quality of care. *Social Sci Med* 2000; 51: 1611-1625. | concept |
| Chassin M, Galvin RW, National Roundtable on Health Care Quality. The urgent need to improve health care quality: Institute of Medicine National Roundtable on Health Care Quality. *J Am Med Assoc* 1998; 280: 1000-1005. | concept |
| Chaudhry B, Wang J, Wu S, et al. Systematic review: Impact of health information technology on quality, efficiency, and costs of medical care. *Annal Intern Med* 2006; 144: 742-752. | concept |
| Chelmowski MK. All-or-none measurement of health care quality. *J Am Med Assoc* 2006; 296: 392-393; author reply 393. | quality in service |
| Chen MK. The K index: a proxy measure of health care quality. *Health Serv Res* 1976; 11: 452-463. | quality in service |
| Clancy CM. AHQRs National Healthcare Quality and Disparities Reports: Resources for health services researchers. *Health Serv Res* 2006; 41: xiii-xix. | concept |
| Cleves MA, Weiner JP, Cohen W, et al. Assessing HCFA's Health Care Quality Improvement Program. *Joint Commission J Qual Improv* 1997; 23: 550-560. | reporting |
| Coates AS. Application of quality of life measures in health care delivery. *J Palliat Care* 1992; 8: 18-21. | concept |
| Cooperberg MR, Birkmeyer JD, Litwin MS. Defining high quality health care. *Urol Oncol* 2009; 27: 411-416. | patient quality measures |
| Damman OC, van den Hengel YK, van Loon AJ, et al. An international comparison of web-based reporting about health care quality: content analysis. *J Med Internet Res* 2010; 12: e8. | quality in service |
| Davila F. What is an acceptable and specific definition of quality health care? *Proceed (Baylor Uni Med Centre)* 2002; 15: 84-85. | concept |
| DesHarnais SI, Forthman MT, Homa-Lowry JM, et al. Risk-adjusted quality outcome measures: indexes for benchmarking rates of mortality, complications, and readmissions. *Qual Manage Health Care* 1997; 5: 80-87. | concept |
| Diamond GA, Denton TA, Matloff JM. Fee-for-benefit: a strategy to improve the quality of health care and control costs through reimbursement incentives. *J Am College Cardiol* 1993; 22: 343-352. | concept |
| Donabedian A. Promoting quality through evaluating the process of patient care. *Med Care* 1968; 6: 181-202. | concept |
| Donabedian A. *A guide to medical care administration, Volume II: Medical Care Appraisal*. American Public Health Association: New York, 1969. | concept |
| Donabedian A. *Explorations in quality assessment and monitoring: the definition of quality and approaches to assessment*. Ann Arbor, Michigan: Health Administration Press, 1980. | concept |
| Donabedian A. The quality of care: how it can be assessed? *J Am Med Assoc* 1988; 260: 1743-1748. | quality in service |
| Donabedian A. The seven pillars of quality. *Arch Pathol Lab Med* 1990; 114: 1115–1118. | concept |
| Druss, B, Rosenheck R. Evaluation of the HEDIS measure of behavioral health care quality. *Psychiatr Serv* 1997; 48: 71-75. | concept |
| Druss B, Rosenheck R, Stolar M. Patient satisfaction and administrative measures as indicators of the quality of mental health care. *Psychiatr Serv* 1999; 50: 1053-1058. | quality in service |
| Early GL, Roberts SR. Defining and improving health care quality. *J Am Med Assoc* 1999; 281: 984-985. | quality in service |
| Egger E. Study of '100 Top Hospitals' poses questions about definition for health care quality. *Health Care Strateg Manage* 1999; 17: 20. | quality in service |
| Eselius LL. Assessing the quality of behavioral health care and health plans using consumer reports and ratings. *US, ProQuest Informat Learn* 2004; **65**. | quality in service |
| Faber M, Bosch M, Wollersheim H, et al. Public reporting in health care: how do consumers use quality-of-care information? A systematic review. *Med Care* 2009; 47: 1-8. | concept |
| Feinwachs D. *Empirically assessing the quality of health care: The correlation of patient perceptions and medical outcomes.* US: University of Minnesota. 1990; **51**. | patient quality measures |
| Fouskakis D, Ntzoufras I, Draper D. Bayesian variable selection using cost-adjusted BIC, with application to cost-effective measurement of quality of health care. *Annal Appl Stat* 2009; 3: 663-690. | quality in service |
| Garcia MTM, De Leon AC, Lana AT, et al. Continuous quality improvement in primary health care - A five year project. *Europ J Public Health* 1999; 9: 131-136. | concept |
| Gardner LA, Snow V, Weiss K, et al. Understanding uptake of continuous quality improvement in Indigenous primary health care: lessons from a multi-site case study of the Audit and Best Practice for Chronic Disease project. *Implement Sci* 2010; 5: 21. | quality in service |
| Glance LG, Osler TM, Mukamel DB, et al. Impact of the present-on-admission indicator on hospital quality measurement: Experience with the Agency for Healthcare Research and Quality (AHRQ) Inpatient Quality Indicators. *Med Care* 2008; 46: 112-119. | patient quality measures |
| Golden WE. Defining and improving health care quality. *J Am Med Assoc* 1999; 281: 984-985. | patient quality measures |
| Gouws E, Bryce J, Pariyo G, et al. Measuring the quality of child health care at first-level facilities. *Social Sci Med* 2005; 61: 613-625. | quality in service |
| Green JH. *A phenomenological study of consumers' definition of quality health care.* Ph.D. thesis. US: The University of Utah: 1995; 122. | patient quality measures |
| Gross PA*,* Braun BI*,* Kritchevsky SB, et al. Comparison of clinical indicators for performance measurement of health care quality: A cautionary note. *Brit J Clinic Governance* 2000; 5: 202-211. | concept |
| Gutteling JJ, de Man RA, Busschbach JJV, et al. Quality of health care and patient satisfaction in liver disease: the development and preliminary results of the QUOTE-Liver questionnaire. *Brit Med Council Gastroenterol* 2008; 8: 25. | quality in service |
| Haddad S, Fournier P, Potvin L. Measuring lay people's perceptions of the quality of primary health care services in developing countries. Validation of a 20-item scale. *Int J Qual Health Care* 1998; 10: 93-104. | patient quality measures |
| Harolds JA, Merrill JK. The physician quality reporting initiative: what is it, will it increase health care quality, and should wide participation be encouraged? *Clinic Nuclear Med* 2011; 36: 118-120. | patient quality measures |
| Harris AH*,* Kivlahan DR*,* Bowe T, et al. Developing and validating process measures of health care quality: an application to alcohol use disorder treatment. *Med Care* 2009; 47: 1244-1250. | quality in service |
| Heinemann A, Gershon R, Fisher WP. Development and Application of the Orthotics and Prosthetics User Survey: Applications and Opportunities for Health Care Quality Improvement. *J Prosthet Orthot* 2006; 18: P80-85. | quality in service |
| Hermann RC. *Improving Mental Healthcare: A Guide to Measurement-Based Quality Improvement.* Arlington, VA, US: American Psychiatric Publishing, Inc.; 2005. | patient quality measures |
| Hermann RC, Leff HS, Palmer RH, et al. Quality measures for mental health care: Results from a national inventory. *Med Care Res Rev* 2000; 57: 136-154. | quality in service |
| Hermann RC, Mattke S, Somekh D, et al. Quality indicators for international benchmarking of mental health care. *Int J Qual Health Care* 2006; 18: 31-38. | quality in service |
| Hermann RC, Palmer RH. Common ground: A framework for selecting core quality measures for mental health and substance abuse care. *Psychiatr Serv* 2002; 53: 281-287. | quality in service |
| Hermann RC, Rollins CK. Quality measurement in health care: A need for leadership amid a new federalism. *Harvard Rev Psychiatr* 2003; 11: 215-219. | quality in service |
| Hibbard JH, Peters E, Slovic P, et al. Making health care quality reports easier to use. *Joint Commission J Qual Improv* 2001; 27: 591-604. | concept |
| Hoodless M, Bourke L, Evans F. Quality of rural primary health care: Including the consumer. *Aust J Primary Health* 2008; 14: 82-88. | quality in service |
| Hughes, R., Aspinal, F., Addington-Hall, J.M., et al. It just didn't work: the realities of quality assessment in the English health care context. *Int J Nurs Stud* 2004; 41: 705-712. | quality in service |
| Hwang K. Mpofu E. *Health care quality assessments. Rehabilitation and health assessment: Applying ICF guidelines.* Mpofu E, Oakland T (eds). New York, NY US: Springer Publishing Co**,** 2010; 141-161. | concept |
| Ix M. Reducing the administrative burden of health care quality reporting. *Find Brief: Health Care Financ Organiz* 2008; 11: 1-4. | reporting |
| Kanouse DE, Spranca M, Vaiana M. Reporting about health care quality: a guide to the galaxy. *Health Promot Pract* 2004; 5: 222-231. | reporting |
| Kearney JF, Russell JD. Health care quality assurance: tracer analyses in quality assessment of ambulatory care provided by two outpatient teaching clinics. *J Manip Physiol Ther* 1992; 15: 141. | quality in service |
| Kearney JF, Russell JD. Reporting about health care quality: a guide to the galaxy. *Health Promot Pract* 2004; 5: 222-231. | reporting |
| Kelley E, Moy E, Stryer D, et al. Health care quality and disparities: lessons from the first national reports. *Med Care* 2005; 43: I1-2. | reporting |
| Kelley ET, Arispe I, Holmes J. Beyond the initial indicators: Lessons from the OECD Health Care Quality Indicators Project and the US National Healthcare Quality Report. *Int J Qual Health Care* 2006; 18: 45-51. | concept |
| Kerr EA, Hofer TP, Hayward RA, et al. Quality by any other name?: a comparison of three profiling systems for assessing health care quality. *Health Serv Res* 2007; 42: 2070-2087. | quality in service |
| Kerssens JJ, Groenewegen PP, Sixma HJ, et al. Comparison of patient evaluations of health care quality in relation to WHO measures of achievement in 12 European countries. *Bulletin World Health Organiz* 2004; 82: 106-114. | patient quality measures |
| Kohli R, Tan JK, Piontek FA, et al. Integrating cost information with health management support system: an enhanced methodology to assess health care quality drivers. *Topic Health Informat Manage* 1999; 20: 80-95. | concept |
| Lai M, Afdhal NH. Health care quality measurement in the care of patients with cirrhosis. *Clinic Gastroenter Hepatol* 2010; 8: 649-650. | concept |
| Lane DS, Kelman HR. Assessment of maternal health care quality: conceptual and methodologic issues. *Med Care* 1975; 13: 791-807. | patient quality measures |
| Lee H, Delene LM, Bunda MA, et al. Methods of measuring health-care service quality. *J Bus Res* 2000; 48: 233-246. | quality in service |
| Leslie D, Rosenheck R. Comparing quality of mental health care for public sector and privately insured populations. *Psychiatr Serv* 2000; 51: 650-655. | quality in service |
| Luce JM, Bindman AB, Lee PR. A brief history of health care quality assessment and improvement in the United States. *West J Med* 1994; 160: 263-268. | quality in service |
| Månsson J, Nilsson G, Bjorkelund C, et al. Collection and retrieval of structured clinical data from electronic patient records in general practice: A first-phase study to create a health care database for research and quality assessment. *Scand J Prim Health Care* 2004; 22: 6-10. | quality in service |
| Marshall M, Klazinga N, Leatherman S, et al. OECD Health Care Quality Indicator Project. The expert panel on primary care prevention and health promotion. *Int J Qual Health Care* 2006; 18: 21-25. | concept |
| Martin M, Paine LA. PSO: an evolving, critical role in health care quality: reporting structure varies from facility to facility. *Healthcare Benchmarks Qual Improv* 2007; 14: 64-67. | reporting |
| Mataria A, Donaldson C, Luchini S, et al. A stated preference approach to assessing health care-quality improvements in Palestine: from theoretical validity to policy implications. *J Health Econ* 2004; 23: 1285-1311. | quality in service |
| Mattke S, Epstein AM, Leatherman S. The OECD Health Care Quality Indicators Project: history and background. *Int J Qual Health Care* 2006; 18: 1-4. | concept |
| McClarey M. Quality measures in health care: have they always been with us? *J Res Nurs* 2009; 14: 291-293. | quality in service |
| McDonald KM, Davies SM, Haberland CA, et al. Preliminary assessment of pediatric health care quality and patient safety in the United States using readily available administrative data. *Pediatr* 2008; 122: e416-425. | quality in service |
| Melichar L. Introduction: Improving health care in America through nursing quality measurement research. *Med Care Res Rev* 2007; 64: 3S-9S. | quality in service |
| Merry MD, Crago MG. The past, present and future of health care quality. Urgent need for innovative, external review processes to protect patients. *Physician Exec* 2001; 27: 30-35. | quality in service |
| Meurer SJ, Rubio DM, Counte MA, et al. Development of a Healthcare Quality Improvement Measurement Tool: Results of a Content Validity Study. *Hospital Topics* 2002; 80: 7. | patient quality measures |
| Millar J. SHEA symposium III. Experience in the field: assessing quality of health care reform in Canada. *Infection Control Hospital Epidemiol* 2001; 22: 589-592. | quality in service |
| Miranda DJ. Health care quality reporting: Changes and challenges. *Health Care Financ Rev* 2007; 28: 1-4. | reporting |
| Mitty E. Hastings Center special report: the ethics of using QI methods to improve health care quality and safety. *J Nurs Care Qual* 2007; 22: 97-101. | quality in service |
| Mohlenbrock WC. Value-based health care, Part 2. The physician imperative: define, measure, and improve health care quality. *Physician Exec* 1998; 24: 47-54. | quality in service |
| Naylor MD. Advancing the science in the measurement of health care quality influenced by nurses. *Med Care Res Rev* 2007; 64: 144S-169S. | quality in service |
| Nelson EC, Homa K, Mastanduno MP, et al. Publicly reporting comprehensive quality and cost data: a health care system's transparency initiative. *Joint Commissionion J Qual Patient Saf* 2005; 31: 573-584. | quality in service |
| Nicolucci A, Greenfield S, Mattke S. Selecting indicators for the quality of diabetes care at the health systems level in OECD countries. *Int J Qual Health Care* 2006; 18: 26-30. | patient quality measures |
| Nolan T, Berwick D: **All-or-none measurement raises the bar on performance.** *J Am Med Assoc* 2006, **295:**1168–1170. | concept |
| O'Kane M. Developing measures to ensure quality health care. Interview by Laurie Hall. *Internist* 1995; 36: 17-18. | concept |
| Palmer RH, Miller MR. Methodologic challenges in developing and implementing measures of quality for child health care. *Ambulat Pediatr* 2001; 1: 39-52. | quality in service |
| Peabody JW, Luck J, Glassman P, et al. The use of clinical vignettes to measure the quality of health care. *Annal Internal Med* 2004; 141: 167. | patient quality measures |
| Petterson I-L. Arnetz BB. Measuring psychosocial work quality and health: Development of health care measures of measurement. *J Occup Health Psychol* 1997; 2: 229-241. | quality in service |
| Pincus HA, Naber D. International efforts to measure and improve the quality of mental healthcare. *Current Opin Psychiatr* 2009; 22: 609. | quality in service |
| Rhoads KF, Konety BM, Dudley RA. Performance measurement, public reporting, and pay-for-performance. *Urolog Clinic North Am* 2009; 36: 37-48. | concept |
| Romano PS. Peer group benchmarks are not appropriate for health care quality report cards. *Am Heart J* 2004; 148: 921-923. | concept |
| Roper WL. Medicare, Medicaid, and health care quality: a focus on quality assessment has become ensconced in the nation's health care system, but it was not an easy task. *Health Affairs* 2005; 24: W5-331-332. | reporting |
| Rubin HR, Pronovost P, Diette GB. The advantages and disadvantages of process-based measures of health care quality. *Int J Qual Health Care* 2001; 13: 469-474. | reporting |
| Sacks JG. Definitions of the Quality of the Medical Care: A Strategy for Segmentation of the Health Care Market. *Health Market Quarterly* 1985; 3: 11. | reporting |
| Schade CP, Brehm JG. Improving the home health acute-care hospitalization quality measure. *Health Serv Res* 2010; 45: 712-727. | quality in service |
| Schaffer WA. The next medical breakthrough: defining a research agenda that helps improve health care quality. *AHIP Cover* 2004; 45: 16-20. | quality in service |
| Sederer LI. Review of 'Improving mental health care: A guide to measurement-based quality improvement'. *Psychiatr Serv* 2006; 57: 1531-1532. | quality in service |
| Serrant-Green L. Uniting health care quality and equity: the challenge of measurement. *Qual Primary Care* 2010; 18: 293-295. | quality in service |
| Shaw I. Assessing quality in health care services: lessons from mental health nursing. *J Advance Nurs* 1997; 26: 758-764. | quality in service |
| Shaw LJ, Miller DD. Defining quality health care with outcomes assessment while achieving economic value. *Topic Health Inform Manage* 2000; 20: 44-54. | quality in service |
| Shepard S. Hospitals working to define quality in health care. *Memphis Biz J* 1990; 11: 22. | concept |
| Sheth SC. Perceptions of health care quality as measured by the modified drain survey. *US, ProQuest Inform Learn,* 2007; 68. | quality in service |
| Simpson RL. The role of IT in health care quality assessment. *Nursing administration quarterly* 2003; 27: 355-359. | patient quality measures |
| Smart DT. Ensuring health care quality: Perspective from a member of NCQA's Committee on Performance Measurement. *Clinical Therapeutics* 1997; 19: 1532-1539. | quality in service |
| Smith DB. The measurement of health care quality: A problem in psychological scaling and social decision theory. *Social Sci Med* 1972; 6: 145-155. | quality in service |
| Smith DP, Jordan HS. Piloting nursing-sensitive hospital care measures in Massachusetts. *J Nurs Care Qual* 2008; 23: 23-33. | quality in service |
| Spath PL. *Innovations in health care quality measurement*. US, American Hospital Publishing: 1989. | quality in service |
| Sprague L. Performance measurement: honing our health care quality tools. *Issue Brief/National Health Policy Forum* 1998; 726: 1-2. | reporting |
| Srebnik D, Hendryx M, Stevenson J, et al. Development of outcome indicators for monitoring the quality of public mental health care. *Psychiatr Serv* 1997; 48: 903-909. | patient quality measures |
| Sutton M, McLean G. Determinants of primary medical care quality measured under the new UK contract: cross sectional study. *Brit Med J* 2006; 332: 389-390. | quality in service |
| Tallia AF. Assessing health care quality using HEDIS 3.0. *New Jersey Med* 1996; 93: 53-55. | concept |
| Torpy JM. Raising health care quality: Process, measures, and system failure. *J Am Med Assoc* 2002; 287: 177-178. | quality in service |
| Torres-Vigil I, Aday L, Reyes-Gibby C, et al. Health care providers' assessments of the quality of advanced-cancer care in Latin American medical institutions: A comparison of predictors in five countries: Argentina, Brazil, Cuba, Mexico, and Peru. *J Pain Palliat Care Pharmacother* 2008; 22: 7-20. | quality in service |
| Trevino JJ. Review of 'Improving mental healthcare: A guide to measure-based quality improvement'. *J Clinic Psychiatr* 2007; 68: 642-643. | patient quality measures |
| Ung D. Special report on reimbursement and health care delivery systems. HCFA publishes health care quality improvement system guidelines for state Medicaid managed care programs. *Health Care Law Newsletter* 1994; 9: 18-23. | quality in service |
| Valentine N, Bonsel G, Murray CJ. Measuring quality of health care from the user's perspective in 41 countries: psychometric properties of WHO's questions on health systems responsiveness. *Qual Life Res* 2007; 16: 1107-1125. | patient quality measures |
| Valentine N, Darby C, Bonsel GJ. Which aspects of non-clinical quality of care are most important? Results from WHO's general population surveys of health systems responsiveness in 41 countries. *Social Sci Med* 2008; 66: 1939-1950. | patient quality measures |
| van Campen C, Sixma H, Kerssens JJ, et al. Assessing patients' priorities and perceptions of the quality of health care: The development of the Quote-Rheumatic-Patients Instrument. *Brit J Rheumatol* 1998; 37: 362-368. | patient quality measures |
| Van Matre JG. All-or-none measurement of health care quality. *J Am Med Assoc* 2006; 296: 392. | quality in service |
| Veroff DR, Gallagher PM. Effective reports for health care quality data: lessons from a CAHPS demonstration in Washington State. *Int J Qual Health Care* 1998; 10: 555-560. | quality in service |
| Vorhaus CB, Enthoven AC. Health care quality management: a status report. *Int Anesthesiol Clinic* 1995; 33: 1-14. | quality in service |
| Werner RM, Asch DA. Examining the link between publicly reporting healthcare quality and quality improvement. *Informed consent and clinician accountability: The ethics of report cards on surgeon performance.* Clarke S, Oakley J. (eds). New York, NY US: Cambridge University Press, 2007; 212-225. | reporting |
| Wyatt MT. An empirical examination of selected difference analysis techniques for the evaluation of healthcare quality assessment data. US: The University of Alabama, 1995; 56. | quality in service |
| Zineldin M. The quality of health care and patient satisfaction: an exploratory investigation of the 5Qs model at some Egyptian and Jordanian medical clinics. *Int J Health Care Qual Assur Inc Leader Health Serv* 2006; 19: 60-92. | concept |
